# Management of Pulp Canal Obliteration—Systematic Review of Case Reports

**DOI:** 10.3390/medicina57111237

**Published:** 2021-11-12

**Authors:** Alexandra Vinagre, Catarina Castanheira, Ana Messias, Paulo J. Palma, João C. Ramos

**Affiliations:** 1 Institute of Operative Dentistry, Faculty of Medicine, University of Coimbra, 3000-075 Coimbra, Portugal; avinagre@fmed.uc.pt (A.V.); castanheira.cata@gmail.com (C.C.); joao.ramos@ipmd.pt (J.C.R.); 2 Center for Innovation and Research in Oral Sciences (CIROS), Faculty of Medicine, University of Coimbra, 3000-075 Coimbra, Portugal; 3 Institute of Oral Implantology and Prosthodontics, Faculty of Medicine, University of Coimbra, 3000-075 Coimbra, Portugal; ana.messias@uc.pt; 4 CEMMPRE—Center for Mechanical Engineering, Materials and Processes, University of Coimbra, 3030-788 Coimbra, Portugal; 5 Institute of Endodontics, Faculty of Medicine, University of Coimbra, 3000-075 Coimbra, Portugal

**Keywords:** pulp canal obliteration, tooth injuries, watchful waiting, tooth bleaching, root canal treatment

## Abstract

*Background and Objectives*: This systematic review aimed to assess the literature focusing on the clinical management of traumatized teeth with Pulp Canal Obliteration (PCO) and propose an updated clinical decision-making algorithm. The present review follows the PRISMA guidelines and was registered on PROSPERO database (CRD42020200656). *Materials and Methods*: An electronic search strategy was performed in Pubmed, EBSCOhost and LILACS from inception to March 2021. Only anterior permanent teeth with PCO due to dental trauma were included. Regarding clinical approaches, only teeth managed with a “watchful waiting” approach, tooth bleaching or root canal treatment (RCT) were included. Quality assessment was performed using the JBI Critical Appraisal Tool for Case Reports. *Results*: Twenty case reports were selected, resulting in a total of 27 patients. The number of traumatized teeth diagnosed with PCO was 33. The “watchful waiting” approach was the most implemented clinical strategy. Discolored non-symptomatic PCO teeth were mostly managed with external bleaching. The prevalence of pulp necrosis (PN) was 36.4%. For teeth diagnosed with PN, non-surgical RCT was performed in 10 teeth and surgical RCT in one tooth. Guided endodontic technique was performed in six of those teeth. *Conclusions*: For discolored non-symptomatic PCO teeth, external bleaching is advocated and the RCT approach should not be implemented as a preventive intervention strategy. Symptomatic PCO teeth should follow regular endodontic treatment pathways. Clinical approach of teeth with PCO should follow a decision-making algorithm incorporating clinical and radiographic signs and patient-reported symptoms.

## 1. Introduction

Traumatic dental injuries (TDI) are a public health problem requiring appropriate diagnosis, treatment planning and follow-up to ensure favourable outcome. Upper central and lateral incisors are the teeth most affected by trauma [1,2]. After a TDI, different dental pulp reactions can occur, such as pulp necrosis, internal resorption or pulp canal obliteration [3,4].

Pulp Canal Obliteration (PCO), also known as calcific metamorphosis, is a sequelae of dental trauma and usually affects the anterior teeth of young adults [5,6]. According to the American Association of Endodontists [7], calcific metamorphosis consists of pulp response to trauma characterized by rapid deposition of hard tissue within the root canal and pulp chamber space. However, the exact physiopathological mechanism of PCO is still unknown [8]. This condition is more frequently identified through tooth discoloration or incidentally in routine radiographs [9,10]. In most cases PCO is clinically recognized at least one year after the injury, in contrast with the three months for pulp necrosis [11]. Hence, this shows the importance of clinical and radiographic monitorization of traumatized teeth over time [12].

Frequently, the affected tooth shows discoloration of the clinical crown that becomes darker than normal adjacent teeth. Yellow discoloration is more frequent, although the color may also change to grey. This is a result of the increased dentine thickness, which leads to a reduced translucency of the crown [9,13]. The extent of calcification as well as the discoloration tends to get worse with time [3]. For instance, Holcomb and Gregory [14] concluded that there seems to be no correlation between the amount of tooth discoloration and the degree of the obliteration. Notwithstanding that, several studies attempted to investigate the relation between grey discoloration of the tooth crown and pulp necrosis and found that tooth discoloration has no diagnostic value regarding the assessment of the pulp condition [9,13]. 

It is accepted that sensibility tests of teeth with pulp obliteration are unreliable [9,13]. While some teeth with PCO show threshold values for the electric pulp test (EPT) higher than teeth with a normal pulp, others are not responsive. This brings difficulties in pulp condition interpretation because a negative response to EPT does not automatically imply pulp necrosis [9]. Based on the results of the study of Oginni et al. [9], teeth with complete pulp obliteration were more non-responsive to EPT than those teeth with partial pulp obliteration.

Usually, calcification of the pulp canal space develops towards the apex, first affecting the pulp chamber and then progressing to the root canal [8]. Therefore, radiographically, the obliteration of the pulp canal space can be classified as partial pulp canal obliteration (PPCO) or total pulp canal obliteration (TPCO) [5]. Despite that, a histological study demonstrated that even when the entire canal space of teeth with PCO seems to be radiographically obliterated, it is possible to detect a portion of the remaining pulp space [15]. Another histological study by Lundberg and Cvek [16] evaluated the pulp of 20 traumatized permanent incisors with reduced pulp space and no clinical or radiographic signs of pathology. No microorganisms were found, and a moderate inflammatory process was seen in only one tooth.

The incidence of PCO depends on the type of luxation injury and the stage of root development [8,17]. Andreasen et al. [11] concluded that the greater the damage to the pulp, the lower the chances of pulp surviving. After luxation injuries, PCO was found to be more common in immature teeth, while pulp necrosis was more prevalent in teeth with complete root formation [11]. Oginni et al. [3] found no statistically significant differences between the frequencies of partial or total pulp canal obliteration and the injury type.

Although pulp necrosis is considered the ultimate complication of PCO, it was an uncommon finding [8]. The incidence of pulp necrosis in permanent teeth with PCO ranged from 1% to 16% after an average observational period from 3.4 to 16 years [11,13,14]. A recent study [9] including 276 teeth with PCO reported 27.2% prevalence of pulp necrosis. Robertson et al. [13] suggested that the risk of developing pulp necrosis in teeth with PCO increases over time, while the accessibility for endodontic intervention becomes more restricted. 

Establishing a treatment plan for a tooth diagnosed with calcific metamorphosis is a difficult assignment [9]. The question arises as to whether an invasive approach should be implemented or a more conservative one, based on watchful waiting, if the tooth is asymptomatic. While some authors recommend endodontic treatment as soon as PCO is diagnosed radiographically [12,15], most of the literature supports that prophylactic endodontics, as a routine treatment approach, is not justified [13,14,18]. Instead, it is recommended that these teeth should be monitored clinically and radiographically, and that root canal treatment should only be initiated following the development of periapical disease or clinical symptoms [5,9]. These considerations are based on the relatively low incidence of pulp necrosis and the overall success rate of nonsurgical RCT in teeth with PCO, which has been shown to be around 80% [18].

Considering that up to 24% of traumatized teeth develop some degree of canal obliteration and the inherent potential resulting discoloration, it is crucial that clinicians are aware of treatment possibilities for these cases [5]. As PCO may lead to a decrease in translucency and a darker crown, these alterations can be a challenge in obtaining an aesthetic outcome in the anterior region [19].

The literature mentions four possible treatment options to manage discoloration: external or vital bleaching; prophylactic RCT followed by internal bleaching combined or not with external bleaching (inside-outside bleaching technique); internal and external bleaching without RCT; and extracoronal full or partial coverage restorations [8].

Nonetheless, commonly teeth with PCO remain healthy and functional, with no clinical symptoms or changes at the periapical area [9,14]. The discoloration of the clinical crown and pulp necrosis are the main sequels of PCO [13].

The present systematic review aimed at the assessment of the literature focusing on the clinical management of traumatized teeth with PCO. Based on the results of this review and on the most recent literature, an updated clinical decision-making algorithm is proposed. 

## 2. Materials and Methods

The present systematic review was conducted according to the PRISMA (Preferred Reporting Items for Systematic Reviews and Meta-Analyses) statement and was registered on PROSPERO database (CRD42020200656).

### 2.1. Focused Question

Initially, a PICO specialized framework was used to define the search strategy considering:

1. Population: Anterior permanent teeth with pulp canal obliteration as a sequel to dental trauma;
2. Interventions: “Watchful waiting” approach, conservative approach with tooth bleaching, surgical or non-surgical root canal treatment;
3. Comparison: Was not applicable in this study;
4. Outcomes: Aesthetics (tooth color), signs and symptoms of pulp and periapical condition.

Other variables, such as stage of root development and apical closure and progression of the pulp calcification were also searched and, if present, described.

This review aimed to answer the following focused question: “What clinical approach should be adopted in teeth diagnosed with pulp canal obliteration after trauma?”

### 2.2. Search Strategy

For the identification of studies to be included in this review, an electronic search strategy was performed for MEDLINE via Pubmed, Dentistry and Oral Sources Database via EBSCOhost and LILACS via Virtual Health Library, up to January 2021, according to the combination of the search/MeSH terms and Boolean operators described in Supplementary Materials (Table S1). There was no restriction regarding the publication year. 

Furthermore, additional records were identified by hand searching through reference lists of articles found in the primary search. 

### 2.3. Eligibility Criteria

This systematic review considered as inclusion and exclusion criteria the following items:

#### 2.3.1. Inclusion Criteria

- Clinical studies (randomized controlled trials, controlled clinical trials, prospective, retrospective or cross-sectional studies, case series and case reports) focusing on anterior permanent teeth with complete or partial pulp canal obliteration as a sequel to dental trauma without restriction of age, gender or sample size and considering all possible treatment options: teeth managed with a “watchful waiting” approach (no treatment implemented); teeth managed with a conservative approach with tooth bleaching; teeth treated with surgical or non-surgical root canal treatment;
- Articles written in English, Portuguese or Spanish.

#### 2.3.2. Exclusion Criteria

- In vitro studies, conference abstracts, editorials, commentaries or review articles;
- Studies dealing with primary teeth;
- Absence of radiograph from the time of PCO diagnosis;
- Absence of pre- and post-photographs in the case of management by bleaching;
- No specification for the reason of obliteration;
- Other than trauma etiology for obliteration;
- Patients submitted to orthodontic treatment before, during or after trauma;
- Teeth with active or treated caries lesions.

### 2.4. Study Selection

After identification and removal of duplicate reports, two review authors (C.C., A.V.) independently screened the titles and abstracts. When studies apparently met the inclusion criteria and when the abstract was not available or was insufficient to correctly assess validity, the full texts were obtained and independently analysed by two authors (C.C., A.V.). When agreement was not obtained, a third author (J.C.R.) was consulted. Finally, studies that did not meet the inclusion criteria were excluded. 

### 2.5. Data Collection and Analysis

Two authors (C.C., A.V.) independently extracted data from the selected studies using standardized data extraction forms. In the case of disagreement, a third author (J.C.R.) was consulted.

The extracted data included: author(s), year of publication, country of origin of the study, study design, age and gender of the patient, tooth position, apical status classification, information about the trauma (type of injury and time elapsed between trauma and PCO diagnosis), associated symptoms and signs (including tooth color, swelling and sinus tract), response to diagnostic tests (pulp sensibility and percussion tests), PCO classification, periapical diagnosis, clinical approach implemented, description of the treatment procedures, follow-up period and the assessed outcomes. 

Owing to the heterogeneity of the case reports, the results could not be statistically assessed and, therefore, meta-analysis was not attempted.

### 2.6. Quality Assessment

The methodological quality of each included study was assessed using the Joanna Briggs Institute (JBI) Critical Appraisal Tool for Case Reports [20]. This tool provides an approach to evaluate the quality of case reports based on eight leading explanatory questions, two of which are mostly relevant to cases of adverse drug events. For this reason, an adaptation of these questions was made, and the quality of each case report was evaluated according to the following 8 parameters: (1) patient’s demographic characteristics, (2) history of trauma, (3) patient’s current clinical condition, (4) diagnostic tests or methods and the results, (5) intervention(s) or treatment procedure(s), (6) follow-up period, (7) outcome and (8) takeaway lessons. For each question there are four possible responses: yes, no, unclear or not applicable.

To summarize the results of the JBI appraisal, we used the tool proposed by Murad et al. [21]. The authors propose the attribution of scores 1 or 2 to each leading question. According to the present specific clinical scenario, questions 3 to 7 were considered more relevant in the context of the review and therefore received score 2. The remaining questions (1, 2 and 8) received score 1. If the case report clearly responded to the leading question, the respective parameter received a “yes” (total score); if the information provided was incomplete or not clear, the parameter received an “unclear” (half score); if it was not possible to find the information, the parameter received a “no” (score of zero). After the judgement of each parameter, the scores were added, and the studies were classified as: high quality (score = 13); medium quality (score 11–12.5); and low quality (score ≤ 10.5).

## 3. Results

### 3.1. Study Selection

Electronic search resulted in a total of 1004 studies. Hand search identified seven potentially relevant records. After removing duplicates, 897 articles remained. The titles and abstracts were screened, and 833 irrelevant studies were excluded. Then, 64 full texts were assessed for eligibility and 44 reports were excluded from the review at this stage. 

**Figure 1:**
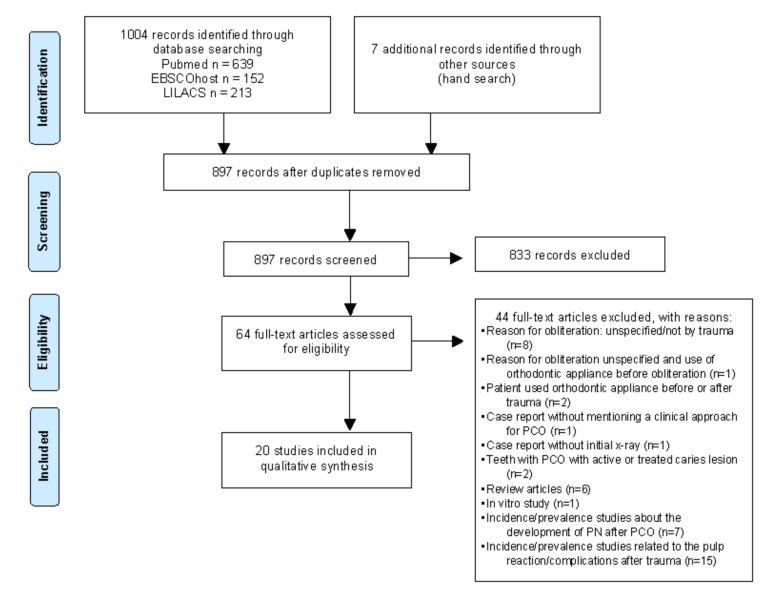
PRISMA flow diagram of systematic searching process.

A total of 20 articles were included [22,23,24,25,26,27,28,29,30,31,32,33,34,35,36,37,38,39,40,41]. Figure 1 describes the selection process. Only case reports were included because studies with a higher level of evidence that addressed treatment options for teeth diagnosed with PCO due to trauma that met the eligibility criteria could not be identified.

### 3.2. Study Characteristics

All selected articles were case reports published between 1985 and 2019. Most of the studies were carried out in Brazil (*n* = 8) and in the USA (*n* = 4), followed by India (*n* = 2) and Germany (*n* = 2). The remaining ones were from Italy, Netherlands, Switzerland and Saudi Arabi. Other characteristics of the included studies, such as demographic data, clinical signs and symptoms and diagnostic tests, are described in Table 1.

### 3.3. Quality Assessment

Methodological quality assessment is described in Table 2. For the 20 case reports included, 2 were evaluated as high quality, 7 as medium quality and 11 as low quality. The studies scored particularly poorly on the following items: patient’s demographic characteristics and history of trauma.

### 3.4. Patient Demographics

The 20 studies described a total of 27 patients ranging from 7 to 51 years, evenly distributed by gender (13 males and 11 females; no data on gender were available for three patients and on age for one patient). 

The total number of teeth diagnosed with PCO included in this analysis was 33. Most of the teeth involved were maxillary incisors (*n* = 31): 17 right central incisors, 13 left central incisors and 1 left lateral incisor. The remaining were mandibular central incisors. At diagnosis, eight teeth were still in the process of root development and apical closure. The majority of studies did not mention the type of injury. 

### 3.5. Signs, Symptoms and Diagnosis

Table 3 describes the pulp and periapical diagnosis, clinical approach and follow-up period and assessed outcomes of the included studies. Periapical radiographs were the most used imaging exams for apical diagnosis. However, CBCT scan was performed on four patients to confirm the presence of apical periodontitis in four teeth with PCO (Cases No. 4, 8, 11, 26). Tenderness to palpation was only evaluated in three patients (Cases No. 22, 24, 27), which resulted in a positive response. 

Based on the data provided by the articles, and through the analysis of the initial periapical radiograph of each tooth, the two authors (C.C., A.V.) classified teeth as: partial pulp canal obliteration (PPCO) when the pulp chamber or root canal was not recognizable or reduced in size; total pulp canal obliteration (TPCO) when both the pulp chamber and root canal were not recognizable radiographically. For the 33 teeth with PCO, 18 had PPCO (54.5%) and 15 showed TPCO (45.5%). The reason for detection of PCO was variable: aesthetics (10 teeth); pain (10 teeth); periodic follow-ups after trauma (10 teeth); incidental finding during routine exams (3 teeth). 

Twelve teeth had PCO associated with pulp necrosis (36.4% prevalence within the sample), six of which presented TPCO. In 83.3% of the referred teeth (*n* = 10), the diagnosis of PN was based on the presence of apical periodontitis in non-responsive teeth to sensibility testing.

At the time of the initial diagnosis, discoloration was not reported in 11 teeth. Eighteen teeth presented discoloration, which was reported as yellow in 10 and not characterized in the remaining. Within these 18 discolored teeth, eight were diagnosed with PN. No color changes were reported in four immature teeth, three of which received a watchful waiting approach.

### 3.6. Clinical Approach, Follow-Up Period and Outcomes

The follow-up period of the studies ranged from immediate (right after the conclusion of the treatment or after PCO diagnosis) to 13 years. The clinical approaches implemented are summarized in Table 4. 

Conservative management was adopted for 19 teeth, of which 12 underwent a “watchful waiting” approach and the other 7 underwent external bleaching. Non-surgical RCT was performed in 12 teeth. Two of them showed discoloration of the crown and were treated prophylactically by conventional access (Cases 3, 18) and the remaining 10 had symptoms or signs of periapical disease. 

The outcomes varied across the studies (Table 3), but all teeth survived during the follow-up period. 

The “watchful waiting” approach was the most implemented strategy. Two teeth presented TPCO and 10 presented PPCO, the last with follow-up periods ranging from 6 months to 12.5 years. None showed evidence of periapical changes. Among PPCO cases, two revealed a slight yellow discoloration of the crown and eight went through a continued calcification of the root canal space. 

For the cases managed with external bleaching, five achieved an aesthetic result immediately after treatment, whereas two underwent a second bleaching process. At the last recall visit of these cases, the aesthetic result was maintained (Cases 16, 19).

In one case (Case 27), guided endodontic technique was chosen as the most appropriate treatment approach after unsuccessful attempts to locate the canal. In a similar situation, the treatment choice was apical surgery (Case 23). 

## 4. Discussion

Pulp canal obliteration is a frequent sequel of dental trauma; thus, it is important to make informed evidence-based treatment decisions when managing teeth with this clinical condition. The present systematic review identified 27 cases of PCO after trauma that were managed with different clinical approaches and degrees of invasiveness.

One major finding of this study was that a significant proportion of teeth with PCO (36.4%), without periapical pathology, were successfully managed with a “watchful waiting” approach, remaining functional and healthy during the follow-up period that ranged from 6 months to 12.5 years. 

Although crown discoloration is a common finding in teeth with PCO [14], tooth color was not assessed in all the studies included, hence it was not possible to correlate this clinical sign to the treatment and corresponding outcome. Even so, when discoloration was mentioned, some case reports referred to different solutions to solve this aesthetic problem. According to several authors, external bleaching should be considered the first clinical option to manage discoloration in teeth with PCO, as it is the most conservative approach, allows tooth structure preservation [32,33,34,35,36], is easy to perform and more affordable than other restorative strategies [29]. However, there is a high heterogeneity observed among the cases managed with external bleaching due to the different follow-up periods and bleaching protocols, the varied number of clinical sessions or daily time use, the different product concentrations, and the use or not of a light-activated system. In fact, not only might the lower permeability of the calcified tissue slow down the bleaching progress and impose longer treatment times [42], it may also colordemand a different whitening protocol after the first bleaching strategy. This is shown in the cases of Ramos et al. [36], Silva and Muniz [40] and Muniz et al. [34]. Notwithstanding that, more studies are required to document the long-term efficacy of this treatment in teeth with PCO. Nevertheless, if an unsuccessful outcome is obtained, there is always the possibility to undergo a more invasive intervention such as the application of direct or indirect resin composite or ceramic restorations [13,42]. 

Internal and external bleaching without RCT is another bleaching protocol described in the literature for teeth without apical pathology, and was performed in the study of Aldaiji and Alsahaly [22]. It consists of preparing an access cavity with the removal of coronal dentine followed by placement of a base on the floor of the access cavity, without considering endodontic intervention. However, other scholars do not support this treatment strategy [8]. In two other studies [24,35], teeth without periapical lesion were submitted to prophylactic non-surgical RCT in order to allow internal bleaching of the discolored central incisor. However, such an approach is only advised whenever there are aesthetic concerns in teeth with indication for RCT [19,42]. Thus, there was no indication for endodontic treatment in these two clinical cases and external bleaching should have been considered as first treatment option, followed by the application of direct or indirect restorations in the case of non-response. 

Several prospective studies aiming to determine the incidence of pulp necrosis after PCO have been published over time [11,13,14]. It seems reasonable to do non-surgical endodontic treatment only in teeth with PN evidenced by periapical pathology and/or clinical symptoms [5,9,13]. Only one case [32] involving a tooth showing a periapical lesion was not endodontically treated because the patient did not develop pain or any other discomfort since the trauma. However, according to the indications mentioned above and to the guidelines of the European Society of Endodontology (ESE), RCT should have been performed [43]. 

The study of Cvek et al. [18] was the only one that investigated the prognosis of non-surgical RCT in teeth with PCO post-trauma. The conclusion was that conventional endodontic treatment of teeth with PCO and periapical disease is associated with a success rate of 80% after four years, with a higher failure rate in lower incisors. However, the rate dropped to 50% in the cases with a technical failure at the time of treatment. Therefore, the presence of technical problems during treatment affects negatively impacts the prognosis of the teeth. For this reason, these complications (such as instruments failing or perforation) should be prevented [8].

Consistent with previous reports [18,44], this study presents several cases that highlight the difficulty of performing RCT in teeth with pulp obliteration. The most mentioned problems relate to the need for significant removal of the tooth structure during conventional access opening [24,30,35] and to the difficulty in locating the root canal [38,41], which can be overcome with several safe and feasible clinical strategies, such as CBCT scans, magnification with microscopy and ultrasonic tips [10]. When conservative attempts to locate the canal are unsuccessful, two other treatment options have been advocated in the literature: RCT with guided access [45] and endodontic (root end) surgery [46,47].

CBCT scans are mandatory for root canal treatment with guided endodontic access. This approach was successfully performed in four maxillary and two mandibular incisors with PCO and apical pathology [25,28,31,41] using small diameter burs for preservation of tooth structure. The absolute quantitative loss of tooth substance associated with the access cavity preparation using conventional techniques or static guided access endodontics in calcified teeth has been evaluated. This new approach allows a more predictable and expeditious location and negotiation of calcified root canals with significantly less tooth substance loss [48,49]. However, the main disadvantage of guided endodontic access is the frequent need to wear down the incisal edge in order to enable a straight-line access, which could not be avoided in three teeth of the above-mentioned cases [25,28]. Moreover, clinicians must take into account others difficulties, such as a greater radiation burden and risk of perforation, higher costs and more demanding visualization [49]. 

Notwithstanding that, the authors were unanimous in mentioning that if the identification of the canal becomes too difficult, referral to a specialist endodontist is recommended [19,46].

Fonseca Tavares et al. [41] mentioned that apical surgery, although being a more invasive approach, can be considered in some cases, for example, in severely curved canals where the guided endodontics cannot be performed. The guidelines of the ESE [43] suggest that when it is not possible to treat the tooth from within the pulp chamber, endodontic microsurgery should be considered. This surgical approach can be successfully completed, as evidenced by one of the cases of Schindler and Gullickson [38]. 

A major strength of the present review is that it only includes PCO cases due to dental trauma, excluding all other suggested etiological mechanisms of PCO, such as caries lesions, restorative procedures and orthodontic treatments [25].

The major limitation of the present review is that data on clinical signs, symptoms and response to diagnostic tests (i.e., percussion and pulp sensibility tests) were missing in several cases, therefore the periapical condition seemed to be the most reliable criterion to diagnose PN. However, it is worth noting that when clinical data were available, almost all teeth showing this endodontic complication were tender to percussion, non-responsive to sensibility tests and had symptoms of pain. 

In addition to the limited available data and frequent incomplete description of the circumstances of the trauma at the initial diagnosis, the follow-up period was often immediate or short-term, which contributed to the impossibility of quantitatively analysing the results and establishing further well-defined associations between treatment and outcome. Reports from future case reports or case series should be standardised using, for example, the CARE guidelines [50], and include longer follow-up periods.

**Figure 2:**
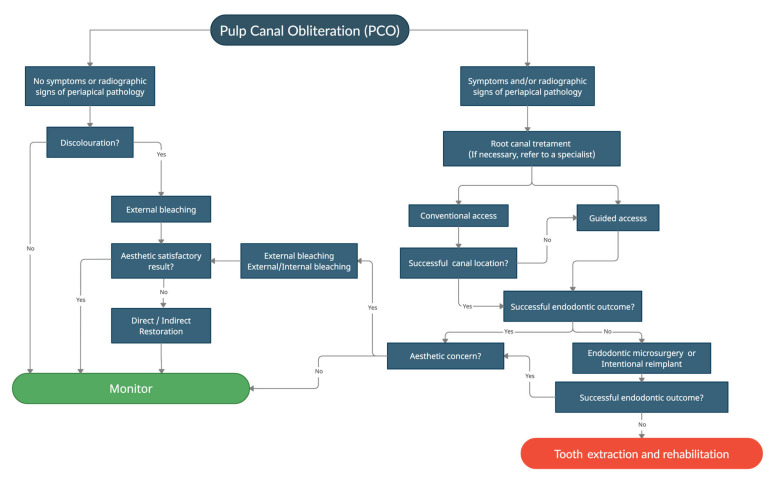
Clinical decision-making algorithm for teeth diagnosed with PCO.

Due to the nature of dental trauma and chronological variability of the clinical establishment of PCO, it is difficult to conduct studies with higher evidence to determine the effectiveness of different clinical approaches and to properly assess the prognosis of these teeth. Thus, the results of the present review were based only on case reports, which are uncontrolled observational studies. This type of study is associated with a high risk of bias, which can be difficult to assess because the authors usually do not report interventions that failed. However, given the unavailability of higher-level evidence that met the inclusion criteria of this study, and since clinicians still need to make treatment decisions for patients showing this condition, a clinical decision-making algorithm (Figure 2) is proposed to make a proper treatment decision in cases of PCO, based on case reports [8,19]. Treatment suggestions are made according to clinical and radiographic signs and symptoms, including signs of discoloration. The endodontic approach was indicated in the presence of symptoms and/or radiographic signs of periapical pathology. If necessary, refer to an endodontic specialist. Depending on the case, clinicians can choose conventional access or, if possible, a more conservative way by the guided access [41,51,52,53,54,55,56,57,58]. Based on the outcomes, in case of failure, endodontic microsurgery is indicated [59] or even an intentional reimplant when the surgery is not possible [60,61,62]. In the last instance, tooth extraction must be considered, followed by a proper rehabilitation procedure.

## 5. Conclusions

Watchful waiting was the most frequent clinical approach implemented in PCO teeth. The literature also suggests that the prophylactic RCT approach should not be used as a preventive intervention or as the first line of action for discolored non-symptomatic PCO teeth. In these cases, external bleaching should be the first strategy to address aesthetic concerns. Symptomatic PCO teeth should follow regular endodontic treatment pathways.

## Figures and Tables

**Table 1:** Characteristics of the included studies, such as demographic data, clinical signs and symptoms and diagnostic tests.

Reference	Country Study Design	Case Number	Age (Y) and Gender of Patient	Tooth (Stage of Root Development)	Trauma		Crown Discoloration (Shade)	Symptoms	Percussion Tenderness	Pulp Sensibility Tests	
					Type of Injury	Time of Injury				Thermal	Electric
Aldaijy and Alsahaly, 2018 [22]	Saudi Arabia Case report	1	36 Female	11	NR	20 y ago	Yes (Cervical: A3.5 Middle: A2)	No	NR	NR	NR
Biagi, 2014 [23]	Italy Case report	2	9 Male	21 (Immature, open apex)	Avulsion	6 m ago	No	No	NR	Pos	
De Cleen, 2002 [24]	Netherlands Case report	3	34 Female	11	Uncomplicated crown fracture	25 y ago	Yes (NR)	NR	NR	NR	NR
Connert et al., 2018 [25]	Switzerland Case report	4	51 Male	31	NR	>30 y ago	Yes (Yellow)	Yes	Yes	Neg	Neg
				41	NR	>30 y ago	Yes (Yellow)	Yes	Yes	Neg	Neg
Gomes et al., 2013 [26]	Brazil Case report	5	8 Male	11 (Immature, open apex)	Intrusion	7 m ago	NR	NR	NR	NR	NR
				21 (Immature, open apex)							
Johnson et al., 1985 [27]	USA Case report	6	8 Male	11 (Immature, open apex)	Avulsion	1 y ago	NR	NR	NR	Neg	
				21 (Immature, open apex)	Intrusion	1 y ago	NR	NR	NR	Neg	
		7	8 Male	21 (Immature, open apex)	Avulsion	7 m ago	NR	NR	NR	Cold: Pos Heat: Neg	Neg
Krastl et al., 2016 [28]	Germany Case report	8	15 Male	11	NR	7 y ago	Yes (NR)	Yes	Yes	Cold: Neg	Neg
Kwon, 2019 [29]	USA Case report	9	NR	21	NR	NR	Yes (Cervical and Middle: D3 Incisal: A2)	NR	NR	NR	Pos
Lakinepally et al., 2018 [30]	India Case report	10	35 Male	11	NR	3 m ago *	Yes (NR)	Yes	Yes	Neg	Neg
Lara-Mendes et al., 2018 [31]	Brazil Case report	11	26 NR	21	NR	13 y ago	NR	Yes	Yes	Neg	Neg
Lise et al., 2014 [32]	Brazil Case report	12	24 Female	11	NR	10 y ago	Yes (Yellow)	NR	NR	Neg	NR
		13	35 Male	21	NR	20 y ago	Yes (Yellow)	NR	NR	Neg	NR
		14	25 Female	11	NR	11 y ago	Yes (NR)	No	NR	Neg	NR
Mourad et al., 2018 [33]	Germany Case report	15	8 Female	11 (Immature, open apex)	Concussion	1 y ago *	No	No	No	Cold: Pos	NR
Muniz et al., 2005 [34]	Brazil Case report	16	48 Female	11	NR	≥12 y ago	Yes (Yellow)	No	NR	Neg	
Raghuvanshi et al., 2015 [35]	India Case report	17	26 Male	21	NR	5 y ago	Yes (NR)	Yes	No	Neg	Neg
		18	21 Male	11	NR	2 y ago	Yes (NR)	NR	No	Neg	Neg
Ramos et al., 2013 [36]	Brazil Case report	19	33 Male	11	NR	>10 y ago	Yes (NR)	No	NR	Pos	Pos
Sacchetto et al., 2011 [37]	Brazil Case report	20	8 Female	21 (NR)	Intrusion	18 m ago	No	NR	NR	Cold: Neg Heat: Neg	NR
Schindler and Gullickson, 1988 [38]	USA Case report	21	10 Female	11	Lateral luxation	6 m ago	NR	No	NR	Cold: Pos	Pos
				21							
		22	48 Male	11	NR	18 y ago	NR	Yes	Yes	Cold: Neg	Neg
		23	35 Male	21	NR	10 y ago	Yes (NR)	No	NR	Cold: Neg	Neg
Shuler et al., 1994 [39]	USA Case report	24	7 NR	11 (Immature, open apex)	Luxation and extrusion	10 m ago	No	Yes	Yes	Cold: Neg	Neg
Silva and Muniz, 2007 [40]	Brazil Case report	25	19 Female	21	NR	NR	Yes (Yellow)	No	NR	NR	NR
Fonseca Tavares et al., 2018 [41]	Brazil Case report	26	43 Female	11	NR	25 y ago	Yes (Yellow)	Yes	Yes	Neg	Neg
		27	24 Female	11	Luxation	>11 y ago	Yes (Yellow)	NR	Yes	NR	NR
				21	Luxation	>11 y ago	NR	No	NR	NR	NR
				22	Luxation	>11 y ago					

m, month(s); y, year(s); NR, not reported; Neg, Negative response; Pos, Positive response; *, second trauma.

**Table 2:** Methodological quality assessment of the included studies. Y—total score (1 or 2); U—half score (0.5 or 1); N—no score (0).

	1—Patient’s Demographic Characteristics	2—Trauma History	3—Patient’s Current Clinical Condition	4—Diagnostic Tests/Methods and Results	5—Intervention(s)/Treatment Procedure(s)	6—Follow-up	7—Outcome	8—Takeaway Lessons	Total Score	Quality
Description	Patient’s Age, Sex and Medical History	Information about the Dental Trauma: Type and Time of Injury	Clinical Signs (Namely Crown Discoloration) and Symptoms	Diagnostic Tests or Methods Used (Imaging Exams, Pulp Sensibility Tests and/or Percussion Tests)	Description of the Intervention or Treatment Protocol in Detail	Follow-up Period	Assessed Outcomes Related to the Aesthetic Result and/or Pulp and Periapical Condition	Key Lessons Learned from the Case		
Score	1	1	2	2	2	2	2	1	13	
Aldaijy and Alsahaly, 2018 [22]	U 0.5	U 0.5	Y 2	U 1	Y 2	U 1	U 1	Y 1	9	Low
Biagi, 2014 [23]	Y 1	Y 1	Y 2	Y 2	Y 2	Y 2	Y 2	Y 1	13	High
De Cleen, 2002 [24]	U 0.5	Y 1	U 1	U 1	U 1	N 0	U 1	Y 1	6.5	Low
Connert et al., 2018 [25]	U 0.5	U 0.5	Y 2	Y 2	Y 2	N 0	Y 2	Y 1	10	Low
Gomes et al., 2013 [26]	U 0.5	Y 1	N 0	U 1	Y 2	Y 2	Y 2	Y 1	9.5	Low
Johnson et al., 1985 [27]	U 0.5	Y 1	N 0	Y 2	Y 2	Y 2	Y 2	Y 1	10.5	Low
Krastl et al., 2016 [28]	U 0.5	U 0.5	Y 2	Y 2	Y 2	Y 2	Y 2	Y 1	12	Medium
Kwon, 2019 [29]	N 0	N 0	U 1	Y 2	Y 2	N 0	Y 2	U 0.5	7.5	Low
Lakinepally et al., 2018 [30]	U 0.5	U 0.5	Y 2	Y 2	Y 2	U 1	Y 2	Y 1	11	Medium
Lara-Mendes et al., 2018 [31]	U 0.5	U 0.5	U 1	Y 2	Y 2	Y 2	Y 2	Y 1	11	Medium
Lise et al., 2014 [32]	U 0.5	U 0.5	U 1	Y 2	Y 2	N 0	Y 2	Y 1	9	Low
Mourad et al., 2018 [33]	Y 1	Y 1	Y 2	Y 2	Y 2	Y 2	Y 2	Y 1	13	High
Muniz et al., 2005 [34]	U 0.5	U 0.5	Y 2	Y 2	Y 2	Y 2	Y 2	Y 1	12	Medium
Raghuvanshi et al., 2015 [35]	U 0.5	U 0.5	Y 2	Y 2	U 1	N 0	U 1	Y 1	8	Low
Ramos et al., 2013 [36]	Y 1	U 0.5	Y 2	Y 2	Y 2	Y 2	Y 2	Y 1	12.5	Medium
Sacchetto et al., 2011 [37]	U 0.5	Y 1	U 1	Y 2	Y 2	Y 2	Y 2	Y 1	11.5	Medium
Schindler and Gullickson, 1988 [38]	U 0.5	U 0.5	U 1	U 1	Y 2	Y 2	Y 2	Y 1	10	Low
Shuler et al., 1994 [39]	U 0.5	Y 1	Y 2	Y 2	Y 2	Y 2	Y 2	U 0.5	12	Medium
Silva and Muniz, 2007 [40]	U 0.5	N 0	Y 2	U 1	Y 2	N 0	Y 2	Y 1	8.5	Low
Fonseca Tavares et al., 2018 [41]	U 0.5	U 0.5	Y 2	Y 2	Y 2	U 1	U 1	Y 1	10	Low

Y, Yes; N, No; U, Unclear.

**Table 3:** Diagnosis, clinical approach, follow-up period and outcomes.

Reference	Case Number	Tooth	Diagnosis		Clinical Approach	Treatment Procedures	Follow-Up	Outcome
			PCO Type	Apical Diagnosis				
Aldaijy and Alsahaly, 2018 [22]	1	11	Total	Normal apical tissues	Internal bleaching without RCT	Walking bleach technique: rubber dam; access cavity; glass ionomer cement base at the CEJ; 3 applications with 1 week interval of 35% hydrogen peroxide gel	2 w	Tooth 11 presented a successful aesthetic result and no evidence of periapical changes
Biagi, 2014 [23]	2	21	Partial	Normal apical tissues	Watchful waiting	Periodic examination	Yearly during the first 5 y 7.5 y 9.5 y 12.5 y	During the follow-up period, tooth 21 revealed continued root canal calcification and root development. After 12.5 years, the tooth showed total PCO, slight yellow discoloration of the crown and no evidence of periapical changes
De Cleen, 2002 [24]	3	11	Total	Normal apical tissues	Prophylactic non-surgical RCT and internal bleaching	Conventional RCT and then walking-bleach technique	Immediate	Tooth 11 revealed sub-obturation and a successful aesthetic result
Connert et al., 2018 [25]	4	31	Partial	Radiolucency (CBCT): Apical periodontitis	Non-surgical RCT	Guided Endodontics: 2 sessions with 2-week intervals; CBCT and intra-oral scan; template; drill Ø0.85 mm; 1% NaOCl; reciprocating file; Ca(HO)_2_ dressing; vertically condensed gutta-percha, epoxy sealer	Immediate	Teeth 31 and 41 showed an adequate RCT
		41	Partial	Radiolucency: Apical periodontitis				
Gomes et al., 2013 [26]	5	11	Partial	Normal apical tissues	Watchful waiting	Periodic examination	7.5 y 9.5 y	During the follow-up period, teeth 11 and 21 revealed complete root formation and continued root canal calcification. After 9.5 years, both teeth showed total PCO without clinical or radiographic signs or symptoms
		21	Partial					
Johnson et al., 1985 [27]	6	11	Partial	Normal apical tissues	Watchful waiting	Periodic examination	6 m	Tooth 11 revealed continued root canal calcification, complete apical closure and no evidence of periapical changes
		21	Partial	Normal apical tissues	Watchful waiting	Periodic examination	6 m	Tooth 21 revealed further apical closure without evidence of periapical changes
	7	21	Partial	Normal apical tissues	Watchful waiting	Periodic examination	1 y 2 y	During the follow-up period, tooth 21 revealed continued root canal calcification and root development. After 2 years, the tooth showed complete apical closure, total PCO, slight yellow discoloration of the crown and no evidence of periapical changes
Krastl et al., 2016 [28]	8	11	Partial	Radiolucency (CBCT): Apical periodontitis	Non-surgical RCT	Guided endodontics: 2 sessions with 4-week intervals; CBCT and intra-oral scan; template; drill Ø1.5 mm; 1% NaOCl; K-file size 10; EAL; rotatory instrumentation system up to 50.04 file; Ca(HO)_2_ dressing; vertically condensed gutta-percha, epoxy sealer	15 m	Tooth 11 showed no clinical or radiographic signs or symptoms of apical pathology
Kwon, 2019 [29]	9	21	Total	Normal apical tissues	External bleaching	Single-tooth in-office bleaching: 2 sessions with 1 week interval, 45 min, 38% hydrogen peroxide gel, gingival resin barrier + single tooth at-home bleaching: 2 weeks, carbamide peroxide gel	Immediate	Tooth 21 presented a successful color matching to the adjacent teeth (In cervical region: B1, Middle: C1, Incisal: A1)
Lakinepally et al., 2018 [30]	10	11	Total	PL space widening: Apical periodontitis	Non-surgical RCT	Conventional RCT: 1 session; US BUC 1 tips; DG 16 explorer; DOM; EAL; crown down technique; 17% EDTA gel; 5.25% NaOCl; K-file size 8, C+ file size 8, ProTaper Next rotatory files up to size X2; ProTaper Next X2 gutta-percha, resin-based sealer	3 m	Tooth 11 was asymptomatic and showed periapical healing
Lara-Mendes et al., 2018 [31]	11	21	Total	Radiolucency (CBCT): Apical periodontitis	Non-surgical RCT	Guided Endodontics: 2 sessions with 14-day intervals; CBCT and intra-oral scan; template; drill Ø1.3 mm; 2.5% NaOCl; EAL; K-file size 10, WaveOne Gold Medium reciprocating; Ca(HO)_2_ dressing; hydraulic compression technique with gutta-percha, epoxy sealer	1 y	Tooth 21 was asymptomatic and showed a small alteration in the PL space
Lise et al., 2014 [32]	12	11	Partial	Normal apical tissues	External bleaching	Single tooth at-home bleaching: 3 weeks, 1 h/day, 10% carbamide peroxide gel	Immediate	Tooth 11 presented a successful color matching to the adjacent teeth
	13	21	Total	Normal apical tissues	External bleaching	Single-tooth in-office bleaching: 9 sessions, 1 h, 3×/week, 37% carbamide peroxide gel without gingival barrier	Immediate	Tooth 21 presented a successful aesthetic result
	14	11	Total	Radiolucency: Apical periodontitis	External bleaching	At-home bleaching: 9 days, 1 h/day, 37% carbamide peroxide gel	Immediate	Tooth 11 presented a successful color matching to the adjacent teeth
Mourad et al., 2018 [33]	15	11	Partial	Normal apical tissues	Watchful waiting	Periodic examination	Every 6 m during the first 2 y 3 y	Tooth 11 was asymptomatic and showed complete apical closure, increased root development, almost complete root canal calcification without evidence of periapical changes or discoloration
Muniz et al., 2005 [34]	16	11	Total	Normal apical tissues	External bleaching	In the first phase, single-tooth in-office bleaching: 6 sessions, 3 applications of 10 min, activation with LED in the first 2 min, 35% hydrogen peroxide gel, rubber dam In the second phase, single-tooth in-office bleaching: 1 session, the same protocol	15 m 30 m	15 months after the first bleaching phase, a slight recurrence of yellowish color in tooth 11 was observed; 30 months after the first phase, tooth 11 presented a successful aesthetic result and no evidence of periapical changes
Raghuvanshi et al., 2015 [35]	17	21	Partial	Sinus tract	Non-surgical RCT	Conventional RCT: 1 session; 17% EDTA; 5.25% NaOCl; K-file size 6, C+ files size 6 and 8, rotatory Protaper files up to F2	Immediate	Tooth 21 showed an adequate RCT
	18	11	Partial	Normal apical tissues	Prophylactic non-surgical RCT	Conventional RCT: 1 session; EDTA gel; K-file size 10 to 25, C+ file size 6 and 8, rotatory Protaper files up to F2; F2 Protaper Gutta-Percha point, epoxy sealer	Immediate	Tooth 11 showed an adequate RCT
Ramos et al., 2013 [36]	19	11	Total	Normal apical tissues	External bleaching	In the first phase, single-tooth in-office bleaching: 1 session, 35% hydrogen peroxide gel, gingival barrier + single-tooth at-home bleaching: 4 h/day, 20% carbamide peroxide gel In the second phase, single-tooth in-office bleaching: 1 session, 2 applications of 15 min, 35% hydrogen peroxide gel, gingival barrier	2 m 5 y	Two months after the first in-office bleaching, a second bleaching session was considered. Five years after bleaching, tooth 11 presented a successful aesthetic result and no evidence of periapical changes
Sacchetto et al., 2011 [37]	20	21	Partial	Normal apical tissues	Watchful waiting	Periodic examination	6 m 18 m 30 m	Tooth 21 revealed no evidence of discoloration nor periapical changes
Schindler and Gullickson, 1988 [38]	21	11	Partial	Normal apical tissues	Watchful waiting	Periodic examination	1 y 2 y 3 y	Teeth 11 and 21 were asymptomatic and showed continued root canal calcification without evidence of periapical changes
		21	Partial					
	22	11	Total	Radiolucency: Apical periodontitis	Non-surgical RCT	Conventional RCT: 2 sessions with 1-week interval; K files; 5.25% NaOCl; laterally condensed gutta-percha, ZOE-based sealer	6 m	Tooth 11 was asymptomatic and showed partial periapical healing
	23	21	Partial	Radiolucency: Apical periodontitis	Surgical RCT	After 2 unsuccessful canal location attempts, an apical surgery was performed. Apical Surgery: Triangular mucoperiosteal flap; retrofilling with amalgam; 4–0 silk sutures	1 y	Tooth 21 was asymptomatic and showed complete periapical healing
Shuler et al., 1994 [39]	24	11	Partial	PL space widening and rarefaction: Apical periodontitis	Non-surgical RCT	Conventional RCT: 1 session; magnification and indirect fiberoptic lighting; EDTA gel; 2.5% NaOCl; K-files up to size 30; laterally condensed and warm gutta-percha, ZOE-based sealer	6 m	Tooth 11 was asymptomatic, revealing complete root development and periapical healing
Silva and Muniz, 2007 [40]	25	21	Total	Normal apical tissues	External bleaching	In the first phase, single-tooth at-home bleaching: 30 days, 6–8 h at night; 16% carbamide peroxide gel In the second phase, single-tooth in-office bleaching only in the cervical region: 2 sessions, 3 applications of 12 min, activation with halogen light in the first 2 min, 35% hydrogen peroxide gel, rubber dam	Immediate	After at-home bleaching, tooth 21 presented a successful color matching to the adjacent tooth in incisal and middle region but a higher saturation in the cervical region. After in-office bleaching, the saturation problem in the cervical region was solved and a successful aesthetic result was obtained
Fonseca Tavares et al., 2018 [41]	26	11	Total	Radiolucency (CBCT): Apical periodontitis	Non-surgical RCT	Guided Endodontics: 1 session; CBCT and gypsum model scan; template; drill Ø1.3 mm; 2.5% NaOCl; EAL; K-file size 15, 30.01 and 30.05 rotatory NiTi Logic System; Tagger’s hybrid technique, resin-based sealer	15 d	Tooth 11 was asymptomatic
	27	11	Total	Radiolucency: Apical periodontitis	Non-surgical RCT	After 1 unsuccessful canal location attempt through conventional RCT, the guided endodontics technique was performed Guided Endodontics: 1 session; CBCT and gypsum model scan; template; drill Ø1.3 mm; 2.5% NaOCl; K-file size 10, 30.01, 25.06, 30.05 and 40.05 rotatory NiTi Logic System; Tagger’s hybrid technique, resin-based sealer	30 d	Tooth 11 was asymptomatic
		21	Total	Normal apical tissues	Watchful waiting	Periodic examination	Immediate	Teeth 21 and 22 were asymptomatic and had no evidence of apical pathology
		22	Total					

Ca(HO)_2_, calcium hydroxide; CBCT, cone-beam computed tomography; CEJ, cementoenamel junction; d, day(s); DOM, dental operating microscopy; EAL, electronic apex locator; m, month(s); min, minute(s); NaOCl, sodium hypochlorite; PL, periodontal ligament; RCT, root canal treatment; US, ultrasonic; y, year(s); ZOE, Zinc Oxide Eugenol.

**Table 4:** Summary of the clinical approaches implemented in teeth with PCO.

Clinical Approach	Number of Teeth (%)
Watchful waiting	12 (36.4%)
External bleaching	7 (21.2%)
Internal bleaching without RCT	1 (3.0%)
Non-surgical RCT	10 (30.3%)
Prophylactic non-surgical RCT	2 (6.0%)
Surgical RCT	1 (3.0%)

## Data Availability

Data will be made available upon request to the corresponding author.

## Supplementary Materials

The following are available online at https://www.mdpi.com/article/10.3390/medicina57111237/s1, Table S1: Search strategy in databases.
