# Supplementary material for: Management of Pulp Canal Obliteration—Systematic Review of Case Reports
Source: Medicina (Kaunas). 2021 Nov 12;57(11):1237. doi: 10.3390/medicina57111237 (PMC8625069; doi:10.3390/medicina57111237)
Supplement: Supplementary file 1 [file medicina-57-01237-s001.zip › medicina-1452715-supplementary.pdf]

Table S1: Search strategy in databases.

**Pubmed search until 2021.01.31:** 639 records<br><br>(“tooth injuries”[MeSH Terms] OR tooth injury OR dental traumatology OR dental trauma OR dental injury OR tooth trauma) AND ("tooth calcification"[MeSH Terms] OR tooth calcification OR “dental pulp calcification”[MeSH Terms] OR dental calcification OR pulp canal obliteration OR pulp obliteration OR pulp calcification OR pulp canal calcification OR dystrophic calcification OR calcific metamorphosis OR “tooth discoloration”[MeSH Terms] OR tooth discoloration OR dental discoloration) AND (treatment OR therapy OR “treatment outcome”[MeSH Terms] OR management OR outcome OR prognosis OR “conservative treatment”[MeSH Terms] OR conservative treatment OR conservative approach OR watchful waiting[MeSH Terms] OR watchful waiting OR wait and see OR tooth bleaching[MeSH Terms] OR bleaching OR whitening OR endodontics OR endodontic treatment OR “root canal therapy”[MeSH Terms] OR root canal therapy OR root canal treatment) NOT ("in vitro techniques"[MeSH Terms] OR “in vitro”)
**EBSCOhost search until 2021.01.31:** 152 records<br><br>(tooth injury OR tooth trauma OR dental trauma OR dental injury OR dental traumatology) AND (tooth discoloration OR dental discoloration OR tooth calcification OR pulp canal obliteration OR pulp obliteration OR pulp calcification OR pulp canal calcification OR dystrophic calcification OR calcific metamorphosis) AND (treatment OR therapy OR management OR prognosis OR outcome OR conservative approach OR conservative treatment OR watchful waiting OR wait and see OR bleaching OR whitening OR endodontics OR endodontic treatment OR root canal treatment OR root canal therapy) NOT “in vitro”<br>Using the Boolean/phrase search mode expanded to related words and equivalent subjects.
**Virtual Health Library search until 2021.01.31:** 213 records<br><br>(mh:("tooth calcification") OR tw:(tooth calcification) OR mh:("dental pulp calcification") OR tw:(pulp canal obliteration) OR tw:(pulp obliteration) OR tw:(pulp calcification) OR tw:(pulp canal calcification) OR tw:(dystrophic calcification) OR

tw:(calcific metamorphosis) OR mh:(“tooth discoloration”) OR tw:(tooth discoloration) OR tw:(dental discoloration)) AND (tw:(dental traumatology) OR mh:("tooth injuries") OR tw:(tooth injury) OR tw:(dental trauma) OR tw:(tooth trauma) OR tw:(dental injury) OR tw:(treatment) OR tw:(therapy) OR mh:(“treatment outcome”) OR tw:(management) OR tw:(outcome) OR tw:(prognosis) OR mh:(“conservative treatment”) OR tw:(conservative treatment) OR tw:(conservative approach) OR tw:(watchful waiting) OR tw:(wait and see) OR mh:("tooth bleaching") OR tw:(bleaching) OR tw:(whitening) OR tw:(endodontics) OR tw:(endodontic treatment) OR tw:(root canal therapy) OR tw:(root canal treatment)) AND NOT (mh:("in vitro techniques") OR tw:("in vitro"))
